# Prenatal Substance Exposure

**DOI:** 10.1146/annurev-devpsych-120621-043414

**Published:** 2023-08-04

**Authors:** Rina D. Eiden, Kristin J. Perry, Miglena Y. Ivanova, Rachel C. Marcus

**Affiliations:** 1 Department of Psychology, The Pennsylvania State University, University Park, Pennsylvania, USA; 2 The Social Science Research Institute, The Pennsylvania State University, University Park, Pennsylvania, USA; 3 Edna Bennett Pierce Prevention Research Center, The Pennsylvania State University, University Park, Pennsylvania, USA

**Keywords:** prenatal substance exposure, prenatal adversity, early adversity, developmental outcomes, pregnancy substance use

## Abstract

This is an evaluative review of the field of prenatal substance exposure, with a focus on neurobiological and behavioral outcomes from infancy to young adulthood. We provide an overall evaluation of the state of the field and comment on current conceptual and methodological issues in need of attention. Although there are many studies of prenatal substance exposure, developmental frameworks that incorporate and reflect the lived experiences of children and families have seldom been employed in this field. In addition, although there are some common effects (e.g., on fetal growth) between major substances, there are also unique effects. Thus, we discuss the role of specific substances but note that polysubstance exposure is common, and models and methods used to date may not be sufficient to advance understanding of coexposure or polyexposure effects. We discuss these conceptual and methodological weaknesses and provide suggestions for future directions.

## PRENATAL SUBSTANCE EXPOSURE

Modern conceptualizations of teratologic effects of substances on fetal development began with the recognition of potential effects of alcohol use in pregnancy on fetal health (for a historical overview, see Sanders 2009), and seminal papers in the early 1970s describing a pattern of effects or a syndrome related to heavy alcohol use (e.g., Jones & Smith 1973). There have been many studies of prenatal substance exposure since then that include almost all major psychoactive substances. Several of these used prospective cohort designs with demographically similar comparison groups, and these have been essential in our understanding of short- and longer-term effects of prenatal substance exposure. Despite these advances, increased prevalence in the use of any substance, or epidemics, have often resulted in a rush to judgement (Mayes et al. 1992), with misleading conclusions that may be harmful to children and families (Mayes et al. 1992) and often disproportionately affect those already facing multiple disparities related to race and income. However, there has been a growing recognition that prenatal substance exposure is complex (Huizink & de Rooij 2018, Schuetze et al. 2021), with challenges related not only to the dose, timing, and chronicity of associations and the role of (epi)genetics, but also to the high prevalence of polysubstance use, prenatal and postnatal comorbidities, and multiple transactional risk and protective factors that relate in important ways to developmental outcomes. Developmental frameworks that consider multiple risks and protective processes transacting across time may be well suited to examining these complexities. We consider these issues in more detail below. Readers are referred to Table 1 for definitions of terminology.

## PREVALENCE AND COMORBIDITY

The prevalence of use of different substances in pregnancy (e.g., alcohol, tobacco, cannabis, cocaine, methamphetamines, phencyclidine, and opioids) has varied enormously over the past two decades. Alcohol and tobacco remain the most-used substances among pregnant women (Tran et al. 2023), with significant demographic variations. Recent (2018–2020) (Gosdin et al. 2022) prevalence of alcohol use in pregnancy is about 13.5%, with about 5.2% reporting binge drinking (four or more drinks on at least one occasion in the past 30 days), a particularly problematic pattern of use that is consistently associated with greater risk for maternal and fetal health (Oei 2020). The prevalence of tobacco use in pregnancy in the form of combustible cigarettes remains high, with rates ranging from 14.9% to 23% nationally and disproportionately higher rates among underserved women with lower levels of education and income (Oh et al. 2017). Tobacco is often co-used with cannabis, with rates of co-use about two-to-three times higher than use of cannabis alone (Haight et al. 2021). This is especially problematic given recent changes in cannabis legalization across the United States, the tripling of cannabis potency over the last few decades, and perceptions that cannabis is safe to use in pregnancy (Arterberry et al. 2019, Bayrampour et al. 2019). Thus, it is not surprising that the rates of cannabis use in pregnancy have also more than doubled over the past decade (Volkow et al. 2019), with current national prevalence estimates of self-reported use ranging from 4.2% to 7% with wide variations across states (Ko et al. 2020a, Volkow et al. 2019). Similarly, there have been dramatic increases in e-cigarette use, cannabis vaping, and opioid use in pregnancy over the past decade (Hirai et al. 2021) with current self-reported estimates of about 7% for prescription pain medication use in pregnancy (Ko et al. 2020b). Rates of other illicit substance use are lower, with cocaine, methamphetamine, and illicit opioids the most-used other substances during pregnancy (Tran et al. 2023). Polysubstance use is common, as noted above, especially among heavier substance users, often making it difficult to attribute exposure effects to any substance alone. There is a small but growing literature on coexposure (e.g., De Genna et al. 2019) and the inclusion of cumulative and proportion (maintaining rank ordering of amount of use) risk scores for exposure as predictors of child outcomes (e.g., Schuetze et al. 2020b). As noted above, there are significant demographic differences in use patterns. For instance, tobacco and cannabis use is more common among younger, non-Hispanic White, unmarried women with lower income and education (e.g., Ko et al. 2020a), while prenatal alcohol use is more common among women with some college education, those who are employed, and those who are not married (Gosdin et al. 2022). Thus, prenatal substance exposure studies need to carefully consider the nature of comparison groups.

## CONCEPTUAL FRAMEWORKS

In addition to polysubstance exposure, prenatal substance exposure is often a marker for other co-occurring pre- to postnatal risks including higher maternal psychological distress and greater partner substance use, violence exposure, and maternal childhood trauma (Ettekal et al. 2020). These additional risk factors may have independent effects on developmental outcomes (for a review, see Schuetze et al. 2021) but may also serve as mediators or have additive effects (Ettekal et al. 2020, Schuetze et al. 2021). Conversely, positive caregiver–child interactions, availability of alternate caregivers, protective community and school contexts, and differences in individual characteristics (intellect, temperament, social orientation, etc.) may all serve as protective factors even in the context of prenatal exposure, although limited studies examine protective processes among substance-exposed children. Given these complexities, there is an urgent need for the field to use developmental frameworks that consider multiple potential mechanisms for risk or resilience and provide a theoretical framework for addressing heterogeneity in outcomes. In a review of the prenatal stress literature, Huizink & De Rooij (2018) concluded that the developmental psychopathology concepts of equifinality (multiple developmental pathways to particular child outcomes) and multifinality (multiple possible outcomes among children exposed to or experiencing the same risk condition) are particularly well-suited to address the complexities in this field. Simultaneously, the behavioral teratology perspective attending to dose, timing, and chronicity effects with careful multimethod and often prospective measurements of exposure has not always been well incorporated in studies addressing equifinality and/or multifinality (Huizink & De Rooij 2018) or the role of genetics (for an exception, see Estabrook et al. 2016). In addition, while there are some common mechanisms (e.g., many substances increase risk for fetal ischemia and hypoxia and impact fetal growth; Bandoli et al. 2019, Graeve et al. 2022, Marchand et al. 2022), substances also have specific effects [e.g., fetal alcohol spectrum disorders (FASDs), withdrawal effects associated with opioid exposure, cleft lip or palate associated with tobacco exposure] that are important to consider in studies utilizing a developmental psychopathology framework. The field is also plagued by difficulties related to accurate measurement of exposure and how to best examine demographic differences in research designs. For instance, in a study of prenatal tobacco exposure, Shisler and colleagues (2017) noted that approximately 23% of families would have been falsely assigned to the nonusing group if measurement had been solely based on self-report. Unfortunately, there is no gold standard measure of exposure, but there is evidence indicating that prospective measures, including well-validated calendar-based interview methods [e.g., the Timeline Followback; Robinson et al. 2014], combined with biomarkers may provide the most accurate estimates of exposure across pregnancy, including the first trimester (Shisler et al. 2017). Finally, animal models are especially helpful in identifying potential mechanisms and in attributing causality to particular substances (Smith 2021), but translation to human development is not always straightforward.

The goal of this review is to summarize exposure effects with these complexities in mind and suggest future directions. There are some excellent reviews of the literature on FASD, the wideranging neurobehavioral, craniofacial, motor, and growth anomalies and outcomes resulting from prenatal alcohol exposure, and we refer the reader to those summaries (e.g., Hanlon-Dearman & Narvey 2022, Mattson et al. 2019). Similarly, prenatal opioid exposure is associated with a constellation of withdrawal symptoms [neonatal opioid withdrawal syndrome (NOWS)] that have been summarized elsewhere (e.g., Patrick et al. 2020). Finally, there are excellent resources with tables on the effects of prenatal exposure to specific substances from NIDA (2020), the American Academy of Pediatrics (Behnke et al. 2013), and Nature Reviews (Thompson et al. 2009). There are also several reviews and meta-analyses on the effects of specific substances that are cited in the current review (e.g., for nicotine, see Bublitz & Stroud 2012; Froggatt et al. 2020; for opioids, see Boggess & Risher 2022, Conradt et al. 2019, Lee et al. 2020, Schuetze et al. 2021; for alcohol, see Hellemans et al. 2010, Henderson et al. 2007, Mattson et al. 2019, Oei 2020; for methamphetamine, see Kunkler et al. 2022, Smith et al. 2015; for cannabis, see Huizink 2014, Scheyer et al. 2019; and for psychoactive drugs, see Salisbury et al. 2009). We refer the reader to these sources for in-depth reviews including tables of effects of specific substances.

We focus this review on a critical appraisal of (*a*) neural, cognitive, and behavioral outcomes of substance exposure; (*b*) contextual risks associated with prenatal exposure that may exacerbate or ameliorate these associations or serve as mediating mechanisms, with a primary focus on parenting; and (*c*) limitations and directions for future research. There is a growing literature on prenatal substance exposure and physical health (e.g., immune function; Dong et al. 2019), but this is not included in the current review. The review is organized by specific substances within each domain of outcomes, followed by an overall summary and future directions.

## NEURODEVELOPMENTAL OUTCOMES

### Prenatal Alcohol Exposure

The effects of in-utero alcohol exposure have been well documented, especially on neurobehavioral outcomes (for an individual participant meta-analysis, see Jacobson et al. 2021) including brain development (e.g., Donald et al. 2015). The literature highlights the importance of dose–response associations with fetal brain structure and function (e.g., Hepper et al. 2012). Although nearly the entire brain is impacted by prenatal alcohol exposure, there are reports of particular abnormalities in the corpus callosum, basal ganglia, and hippocampi, which are linked to verbal and executive function and motor deficits (Lebel et al. 2011). Animal studies have been particularly beneficial in elucidating timing and dose–response effects on neurological functioning. Results indicate that high alcohol concentration is more strongly related to poor brain growth compared with low levels of continuous alcohol exposure (Mattson et al. 2019), and alcohol exposure at any trimester impacts the prefrontal cortex and hippocampus in rats (Brocardo et al. 2017). This is congruent with the human literature, which suggests that alcohol exposure is harmful at any trimester, but first-trimester exposure may have the greatest impact on underlying brain structures (Mattson et al. 2019). However, results are less consistent at low levels of alcohol exposure, although recent results from the Adolescent Brain Cognitive Development Study indicated that children with low prenatal exposure (average of one drink per week) had differences in white matter relative to well-matched controls (Long & Lebel 2022).

Results from an individual participant meta-analysis of six longitudinal cohorts with prospective measures of alcohol exposure and propensity score matching for co-occurring risks indicated modest, but significant and long lasting (into young adulthood), effects of prenatal alcohol exposure on IQ, learning and memory, executive function, reading, and math achievement (for an individual participant meta-analysis, see Jacobson et al. 2021) but no significant associations with sustained attention. Notably, the effect sizes were similar across all five cognitive domains included in the meta-analysis, leading the authors to suggest that there may be underlying common brain structural and functional processes that may be impacted by alcohol. In addition, effect sizes were smaller for the cohort that included middle-class families who may have been able to provide greater environmental enrichment in the postnatal period, highlighting the potential protective effect of the postnatal environment in prenatally exposed cohorts. Behavioral issues may elevate this risk. For instance, in the presence of elevated behavior problems, prenatal alcohol exposure was associated with significant impairments in executive functioning in middle childhood and adolescence (Doyle et al. 2019). As with brain development, results are most consistent for chronic, heavy alcohol use or binge drinking and less consistent for lower levels of exposure (Henderson et al. 2007). Yet, few studies have examined the potentially complex interplay of pre- and postnatal risk and protective factors when considering prenatal alcohol exposure associations across development. In addition, studies have rarely examined trajectories of use across pregnancy (for an exception, see Bandoli et al. 2019), although evidence from this and other substance exposure studies (e.g., Eiden et al. 2013) indicates substantial individual differences in trajectories of substance use during pregnancy. Thus, neurodevelopmental effects at low-to-moderate levels of exposure may be better understood using prospective designs that include other important environmental factors while examining use patterns across pregnancy.

### Prenatal Tobacco and Cannabis Exposure

There is an extensive literature on prenatal tobacco exposure in the form of combustible cigarettes. Much of this literature has significant methodological limitations including poor exposure measurement (often one-to-two-item retrospective reports) and inadequate comparison groups that do not consider the role of poverty or other demographic factors (Estabrook et al. 2016). Despite these limitations, the preponderance of evidence from longitudinal studies with well-validated prospective measures of exposure (e.g., Cornelius et al. 2011) as well as meta-analytic (Froggatt et al. 2020) and systematic (Bublitz & Stroud 2012) reviews suggests significantly lower scores on behavioral measures of attention and orientation among tobacco-exposed infants compared with nonexposed infants. Researchers suggest that these findings may be the result of tobacco exposure being associated with reduced volume in the frontal lobe, lateral ventricular system, and cerebellum. However, meta-analytic reviews indicate significant heterogeneity in outcomes (Froggatt et al. 2020) and emphasize the need for more consistency in findings to understand the impact on underlying brain structures (Bublitz & Stroud 2012). This heterogeneity highlights the role of other co-occurring risk and protective factors and potential differences based on dose, timing, and chronicity of exposure.

Prenatal tobacco exposure has been associated with lower response inhibition and selective attention (Cornelius et al. 2011) and smaller gray matter volume and total brain volume at school age (El Marroun et al. 2014). A few studies have also included mothers’ secondhand-smoke exposure in their conceptualizations of prenatal tobacco exposure and have found an effect of even low levels of prenatal secondhand-smoke exposure on deficits in children’s task initiation and metacognition (e.g., Oh et al. 2020). Increased cortical thinning was also observed for school-age children postnatally exposed to tobacco (for most children, this reflects the chronicity of pre- to postnatal exposure or at least use before pregnancy recognition), which in turn was associated with affective problems (El Marroun et al. 2014), underscoring the importance of examining continued postnatal tobacco use. In the same sample and age range, cannabis-exposed children, most of whom were also exposed to tobacco, had a thicker prefrontal cortex relative to children exposed to only tobacco and control children, potentially reflecting differences in brain maturation (El Marroun et al. 2014).

There are also consistent reports of tobacco exposure effects on brain function, with neural measures indicating impairment in processes related to inhibition (Boucher et al. 2014) and greater activation of several brain areas during these tasks (Longo et al. 2013). However, these neural differences are not always evidenced in behavioral performance, although some of these studies (Longo et al. 2013) had extremely small samples of exposed and nonexposed children, which likely reduced the power to detect behavioral differences. Taken together, results suggest that tobacco-exposed children may have to compensate with greater brain activation to achieve the same behavioral and cognitive outcomes, and long-term effects of such compensation are unclear. There are few recent prospective cohorts with prenatal cannabis exposure (for a low sociodemographic risk sample recruited in the 1970s, see Porath & Fried 2005; for a high sociodemographic risk sample recruited in the 1980s, see Day et al. 1994; and for a mostly low sociodemographic risk sample recruited in the early 2000s, see Jaddoe et al. 2012), and the literature on child outcomes is limited and inconsistent (for a review, see Huizink 2014). Despite the limited work, researchers have theorized that prenatal cannabis exposure impacts the developing fetal endocannabinoid system, which unfolds across age, affecting neurological and cognitive outcomes over time (for reviews, see Huizink 2014, Scheyer et al. 2019). It is also hypothesized that prenatal cannabis exposure is associated with hypoconnectivity in brain areas related to visual-spatial and motor abilities and language production for neonates (Grewen et al. 2015). This is consistent with deficits in verbal and memory function observed in the preschool years (Scheyer et al. 2019). In contrast, other research has found that prenatal cannabis exposure alone is not strongly associated with preschoolers’ executive functioning when comparing any versus no exposure (Murnan et al. 2021). Measurement of the dose and amount of cannabis exposure is particularly challenging with multiple modes of use, variations in potency, and simultaneous use with other substances, making dose–response studies difficult to conduct; yet, this task is of high importance given dramatic increases in the prevalence of cannabis exposure related to increased perceptions of safety and legalization changes (Arterberry et al. 2019, Bayrampour et al. 2019).

### Prenatal Cocaine Exposure

Prenatal cocaine exposure is a marker of polysubstance exposure, and while studies discussed here include careful measurement of other substance use and statistical controls for these other exposures, it is difficult to understand cocaine-specific effects outside of the nested nature of multiple exposures (Eiden et al. 2023). Results are mixed, with some studies reporting little evidence of associations with global measures of cognition (for a review, see Behnke et al. 2013), while others report associations with specific cognitive domains. For instance, in a well-designed longitudinal study with a multimethod measurement of exposure and demographically similar comparison group, cocaine-exposed infants had poorer visual recognition memory (Singer et al. 2005) and a dose–response association with reduced cognitive processing efficiency and executive function difficulties in middle childhood (Minnes et al. 2014). Studies using functional imaging during rest and during a working memory task with emotional distractors suggest that cocaine exposure may increase arousal and alter the balance of excitatory and inhibitory mechanisms processing how cognitive resources are allocated (Li et al. 2011). This is echoed in seminal research on arousal-modulated attention, indicating that unlike non-cocaine-exposed neonates who varied in preferences for stimulation based on their arousal, neonates with prenatal cocaine exposure preferred higher amounts of stimulation regardless of arousal levels (Karmel & Gardner 1996). Emotional arousal may also interfere with performance on cognitive tasks at older ages, such that during an executive function task with emotionally arousing images as distractors, cocaine-exposed adolescents demonstrated altered activity in the ventral prefrontal cortex and in its connection with the amygdala, both relevant for suppressing emotional arousal (Li et al. 2013). Therefore, individuals with prenatal cocaine exposure may have to rely on different brain structures or use more neural resources to suppress emotional responses during everyday cognitive tasks. The long-term impact of these brain alterations is unknown, but some evidence suggests that children with cocaine exposure exhibit greater age-related improvements than those without exposure, narrowing the gap between exposed and nonexposed children (Bridgett & Mayes 2011, Willford et al. 2018).

Others have reported sex differences in cocaine-specific associations. For instance, exposure was associated with poorer short-term memory, lower IQ and verbal reasoning (Bennett et al. 2002), and deficits in central processing and abstract thought for male but not female children (Delaney-Black et al. 2004). Prenatal exposures to cocaine and/or heroin have been associated with attentional problems that impair school-aged children’s ability to attend to visual stimuli, but only for children in maternal care where there may have been high rates of continued postnatal illicit substance use (Ackerman et al. 2008), underscoring the importance of considering the broader context. In addition, these cognitive and neurological effects seem to disappear in young adulthood (e.g., Willford et al. 2018), especially when considering other substance exposures, highlighting the importance of long-term longitudinal studies.

### Prenatal Methamphetamine and Opioid Exposure

A recent meta-analysis of prenatal methamphetamine exposure identified 38 studies, the majority of which were cross-sectional or had weak measurement of exposure, reflecting the state of this literature (Kunkler et al. 2022). The review suggested mixed evidence of methamphetamine exposure effects on brain structure and function, with some indication that striatal regions of the brain may be particularly affected. Meta-analytic results, including findings from one of the few well-designed prospective studies (Infant Development, Environment, and Lifestyle) with multimethod exposure measurement and adequate sample size (Smith et al. 2015), indicated lower global cognitive performance and problem solving ability, poorer short-term memory, and lower language scores among exposed children but with significant heterogeneity and age-related differences in outcomes (Kunkler et al. 2022). Similar to cocaine, polysubstance exposure is common, with alcohol, tobacco, and marijuana use co-occurring with methamphetamine.

There have also been dramatic increases in prenatal opioid exposure through several waves of the opioid epidemic and because adequate treatment for opioid dependence involves Opioid Agonist Therapy (OAT, also called Medication Assisted Treatment) (Jones et al. 2012). As with methamphetamines, the opioid literature has suffered from small sample sizes, cross-sectional designs, and inadequate consideration of demographic differences and polysubstance exposure (for a review, see Schuetze et al. 2021). These weaknesses have been compounded by difficulties in measurement, with variations in the types of opioids used, prescription versus illicit opioid use, and variations within those categories (e.g., buprenorphine and methadone prescribed for OAT, heroin and/or fentanyl often used in the same pregnancies) and additional variations in potency or dose. From the perspective of understanding teratogenic effects, these are complex issues to address. Despite these difficulties, emerging evidence from both human and animal models suggests that prenatal opioid exposure impacts several developing cellular and structural brain functions (for a review, see Boggess & Risher 2022). Brain areas such as the basal ganglia, thalamus, and cerebellar white matter, which have been linked to motor control and sensory deficits, were smaller for independent samples of infants and children aged 10–14 (Boggess & Risher 2022). School-aged children had greater activation of the prefrontal cortex, indicating that they may exert more neural resources for reduced performance (Boggess & Risher 2022). However, while a meta-analysis indicated lower cognitive, psychomotor, and language scores among exposed children (birth to 12 years) (Lee et al. 2020), many effects often disappeared or reduced in magnitude after controlling for sociodemographic, biological, and environmental factors (Schuetze et al. 2021).

## PHYSIOLOGICAL OUTCOMES

Recent theoretical papers have addressed prenatal substance exposure as a marker of toxic fetal stress (e.g., Horn et al. 2018). Employing a translational neuroscience perspective, it has been posited that prenatal substance exposure is one type of teratogenic exposure that influences fetal programming of the hypothalamic-pituitary-adrenal (HPA) axis, immune system response (Horn et al. 2018), and autonomic nervous system (Schuetze et al. 2021). Moreover, prenatal substance exposure influences autonomic nervous system functioning both during rest and in response to stress (for reviews, see Propper & Holochwost 2013, Schlatterer & du Plessis 2021). Below, we summarize the impact of prenatal substance exposure on the autonomic nervous system and HPA-axis functioning.

### Prenatal Alcohol Exposure

Associations between prenatal alcohol exposure and autonomic or HPA reactivity have been mixed, with type of dysregulation (e.g., at rest or baseline versus in response to a stressor or reactivity) varying by study (Hellemans et al. 2010, Reid & Petrenko 2018). Some studies have noted autonomic nervous system and HPA dysregulation among infants and adolescents with prenatal alcohol exposure. For example, exposed infants demonstrated decreased cortisol levels and no changes in respiratory sinus arrythmia (RSA), a marker of parasympathetic activity, in reaction to a physical stressor, compared with nonexposed infants who demonstrated expected RSA decreases and no change in cortisol (Oberlander et al. 2010). Research from animal models indicates that prenatal alcohol exposure may impact HPA responsiveness through interactions between the serotonin and HPA-axis systems (e.g., Hellemans et al. 2010) and alterations in glucocorticoid signaling in the placenta and fetal brain (Lan et al. 2017), resulting in increased HPA activity throughout the life span (Hellemans et al. 2010). Moreover, there is evidence from animal models of sex differences, with gonadal hormones mediating the association between alcohol exposure and HPA-axis activity (Hellemans et al. 2010). This is mirrored in human studies, with boys showing greater cortisol changes during the Still Face procedure relative to girls (Hellemans et al. 2010). However, given that human studies are often underpowered to examine sex differences, prospective studies with strong measurements of alcohol exposure are needed to understand how these processes unfold for humans.

### Prenatal Cannabis and Tobacco Exposure

Relative to alcohol, there is more evidence to support the impact of prenatal cannabis and tobacco exposure on physiological dysregulation. Higher levels of first-trimester tobacco exposure have been associated with autonomic regulation disruptions (Cerritelli et al. 2021). In one prospective cohort recruited in the first trimester of pregnancy [Growing Up Healthy (GUH) Study] (Eiden et al. 2020, Schuetze, et al. 2011), infants with prenatal tobacco and tobacco–cannabis coexposure had a maladaptive increase in RSA in response to frustration, whereas nonexposed infants, particularly boys, demonstrated the expected decrease (Schuetze et al. 2013). Regarding the HPA axis, consistent sex differences have been noted, similar to those with alcohol exposure. For example, in the neonatal period, males but not females coexposed to cannabis and tobacco had lower cortisol values at rest relative to those who were exposed to only tobacco or nonexposed (Stroud et al. 2020). In infancy, GUH data indicated that male, but not female, infants with prenatal tobacco exposure had lower cortisol levels in response to a frustration task compared with nonexposed children (Eiden et al. 2015); in kindergarten, coexposed children demonstrated flatter cortisol responses to frustration relative to nonexposed children (Eiden et al. 2020). In other samples, tobacco-exposed school-aged children had higher cortisol concentrations in hair samples, potentially suggesting chronically high HPA-axis activation (Petimar et al. 2020); however, these associations did not persist into early adolescence. Longitudinal studies of autonomic and HPA activity changes related to exposure and co-occurring risks with a multimethod measurement of exposure and sufficient sample size to examine sex moderation are needed to better understand these outcomes.

### Prenatal Cocaine Exposure

Cocaine exposure may alter physiological systems in multiple ways, including direct effects on the neurotransmitter systems responsible for arousal and stress regulation, increasing fetal stress due to hypoxia and ischemia, and/or epigenetic alterations of stress-arousal regulation systems (Lester & Padbury 2009, Salisbury et al. 2009). Results on autonomic regulation from longitudinal studies with multimethod assessments of exposure are limited and restricted primarily to two samples, the Maternal Lifestyles Study (MLS) (Lester et al. 2016) and the Maternal and Child Health Study (MCHS) (e.g., Schuetze & Eiden 2006). Results from the MLS did not indicate consistent associations between cocaine exposure and autonomic outcomes. However, results from the MCHS with manual editing of movement artifacts in autonomic measures indicated consistent associations between cocaine exposure and RSA that were moderated by child sex. In early infancy, there were dose-dependent associations between cocaine exposure and RSA, with infants of heavier users having lower RSA during sleep (Schuetze & Eiden 2006) and no RSA suppression in response to frustration in later infancy, unlike demographically similar comparison infants (Schuetze et al. 2007). These associations were primarily evident for boys at toddler age (Schuetze et al. 2009), and differences in RSA baseline and reactivity at school age varied by child sex (cocaine-exposed boys displaying higher baseline RSA and cocaine-exposed girls displaying greater reactivity to frustration) (Schuetze et al. 2020a). Perhaps differences in results between the two studies were related to differences in the context of measurement and in how autonomic data were edited for artifacts (an automated process in the MLS versus manual editing in the MCHS). Studies examining associations with HPA reactivity are also limited, with some indication of greater reactivity among cocaine-exposed boys compared with girls in infancy (Eiden et al. 2009), lower cortisol response (using a cortisol difference score from baseline to peak response) among cocaine-exposed early adolescents (Lester et al. 2010), flatter or blunted patterns in response to acute stress among more heavily cocaine-exposed compared with non-cocaine-exposed older adolescents (Chaplin et al. 2015), and no direct associations between cocaine exposure and early adolescent stress reactivity (Eiden et al. 2023).

### Prenatal Exposure to Other Substances and Polysubstance Exposure

Opioid exposure, including treatment for opioid use disorders (e.g., OAT), has acute effects on reductions in fetal heart rate and heart rate variability across pregnancy (for a review, see Conradt et al. 2018). Autonomic symptoms are also a key feature of NOWS, and withdrawal from opioids influences several neurotransmitters that are implicated in stress responses (Conradt et al. 2018). There is evidence of longer-term impacts on physiological functioning, with 6-week-old infants exposed to methadone who received treatment for NOWS demonstrating self-regulation difficulties (Conradt et al. 2018). Additionally, there may be enhanced risk when there are multiple exposures. Results from the MLS indicated that in infancy, cocaine- and opiate-coexposed infants had the largest autonomic response to a sustained attention task relative to infants exposed to only opiates, cocaine, or other substances (Conradt et al. 2014). From 3–6 years of age, higher cumulative prenatal polysubstance exposure was associated with higher levels of baseline RSA and greater RSA reactivity to an attention-demanding task (Conradt et al. 2014). Finally, a study examining group differences in cortisol reactivity between polysubstance-exposed (including cocaine and heroin) and nonexposed adolescents reported less reactivity in the exposed group (Buckingham-Howes et al. 2016).

Importantly, physiological systems do not exist in isolation, and concordance or discordance among the systems may be an important predictor of functioning. For example, discordance among RSA and cortisol levels may be a risk factor for executive dysfunction, which may in turn lead to risky outcomes (Conradt et al. 2014). Additionally, there is an emerging literature on the dual influences of the sympathetic–parasympathetic branches, and therefore, we echo calls from other researchers (e.g., Buss et al. 2018) that future research should examine these dual influences in predicting child outcomes. Future research may also focus on fetal physiological development in the context of prenatal substance and other co-occurring exposures (Conradt et al. 2019).

## BEHAVIORAL OUTCOMES

There is a relatively robust literature on child temperament and behavior problems related to prenatal substance exposure, with ample evidence for potential etiologic associations between temperament and behavior problems, particularly under conditions of high contextual risks (see Gartstein & Skinner 2018). However, only a handful of studies have used methods other than caregiver reports (e.g., observational measures of reactivity and regulation), with fewer measuring behavior across contexts. We highlight some major findings below, with the caveat that this literature has similar complexities and weaknesses as that discussed in the section titled Neurodevelopmental Outcomes.

### Prenatal Alcohol Exposure

There are consistent and long-lasting associations between heavy, chronic alcohol exposure or binge drinking and child behavior problems across development, including conduct disorder, depression, and anxiety, as well as problems with attention, visual perception, self-regulation, and adaptive functioning (skills needed for daily living), that may be present even in the absence of the facial features associated with FASD (Coles et al. 2020) or a FASD diagnosis (Mattson & Riley 2000, Mattson et al. 2019) and using genetically informed designs (Knopik et al. 2009). These associations may be mediated via more difficult temperament in infancy (Gartstein & Skinner 2018). However, as with neurobehavioral outcomes, dose–response associations at lower levels of exposure are not consistent. In addition, it has been suggested that the relationship between alcohol exposure and attention-impulsivity may instead be explained by maternal polysubstance use during pregnancy or environmental factors post-pregnancy (D’Onofrio et al. 2007), although numerous studies examining associations with lower doses used retrospective reports with inadequate measures of dosage. Finally, alcohol-exposed adolescents initiated alcohol use at an earlier age (De Genna et al. 2007), and young adults with prenatal exposure to alcohol but not tobacco were more likely to have alcohol problems (Baer et al. 2003).

### Prenatal Tobacco and Cannabis Exposure

One of the most consistent effects of prenatal tobacco exposure is on externalizing behavior problems beginning in early childhood (e.g., Wakschlag et al. 2006a), with conduct disorder in later childhood (e.g., Cornelius et al. 2007) and higher risk for substance use problems and antisocial behaviors in adolescence and adulthood (Cornelius et al. 2000, De Genna et al. 2017, Wakschlag et al. 2006b). Links with conduct disorder are also consistent among studies using genetically informed designs and well-validated prospective measures of exposure (Estabrook et al. 2016). Some studies suggest a threshold effect of 10 cigarettes or more on these aspects of child functioning (e.g., De Genna et al. 2017). However, developmental processes or mechanisms linking tobacco exposure and externalizing behaviors are not well understood, although there is some suggestion of tobacco’s effects on increased temperamental anger (Gartstein & Skinner 2018), higher reactivity and lower regulation via its effects on fetal growth (Schuetze et al. 2018), and higher effortful control in the context of high arousal, with stronger associations for boys (Wiebe et al. 2015). Results indicate that effortful control at early-school age may partially mediate the association between tobacco exposure and school-age disruptive behaviors (Clark et al. 2016).

Unlike the multitude of studies on prenatal tobacco exposure (many with significant measurement problems), the literature on prenatal cannabis exposure and child behavioral outcomes is more limited. Across cannabis-exposed cohorts varying in sociodemographic risk, results indicated consistent associations between cannabis exposure and higher child impulsivity and hyperactivity (for a review, see Huizink 2014) and, more recently, with higher child aggression and attention problems (Murnan et al. 2021). There are also suggestions of associations due to shared genetic vulnerability given the associations between both maternal and paternal cannabis use and child externalizing problems (El Marroun et al. 2019), although socialization experiences may also play a role given associations between substance use and caregiving risks (see section titled Contextual Factors).

In addition, as noted above, cannabis use often occurs in the context of tobacco use (co-use), and both substances are sometimes used simultaneously (e.g., in the form of blunts). The literature on co-use is limited, and the inclusion of potential additive effects of both substances in analytic models is often missing (De Genna et al. 2019). Using an elegant case-control design with repeated neonatal assessments across the first month of life, there were 42–75% stronger associations between co-use and neonatal attention and lethargy compared with tobacco exposure alone (Stroud et al. 2018). Co-use was also associated with higher autonomic dysregulation in infancy, which in turn was associated with more emotional dysregulation at toddler age (Eiden et al. 2018a), higher infant reactivity, and lower regulation via effects on fetal growth (Schuetze et al. 2018). Co-use was also associated with higher internalizing and sleep problems among girls at preschool age (Eiden et al. 2018b) and blunted stress reactivity patterns at school age compared with tobacco exposure alone (Eiden et al. 2020).

Sex moderation of exposure effects has been noted in several studies, although the direction of the association is not consistent, and few studies examine the role of child sex or gender as a moderator (for a review, see Coles et al. 2012). Associations between tobacco exposure and conduct disorder seem to be stronger for boys (Coles et al. 2012), but there is some evidence of stronger associations between tobacco or cannabis exposure and higher anxiety, depression, attention problems (Eiden et al. 2018b), and internalizing problems generally (El Marroun et al. 2011) among girls compared with boys.

Both tobacco and cannabis have the potential for secondhand exposure during both pregnancy, via maternal exposure to secondhand smoke, and the postnatal period. Secondhand-smoke exposure may be particularly prevalent in low-income samples, with one study indicating that up to 71% of urban, low-income women screened during pregnancy who were not smokers themselves had a household member (most often a partner) who smoked (Eiden et al. 2011c). The literature on secondhand postnatal exposure has not always considered the role of prenatal exposure and vice versa. However, in models accounting for prenatal exposure, continued postnatal exposure was associated with higher toddler emotion dysregulation for boys but not girls (Eiden et al. 2018a), higher infancy exposure (controlling for prenatal exposure) predicted higher child behavior problems at toddler age (Eiden et al. 2018b), and there is evidence for additive effects with prenatal exposure on child behavior problems in middle childhood (Rückinger et al. 2010). These associations between parent substance use and child behavior problems may also be bidirectional, with more behavior problems at toddler age associated with higher maternal cannabis use a year later (Eiden et al. 2018b).

Finally, prenatal tobacco exposure has been linked to higher rates of smoking in adolescence via continued postnatal exposure (Cornelius et al. 2005) and to nicotine dependence in young adulthood (Cornelius et al. 2012). In a predominantly middle-class sample, Porath & Fried (2005) also found that, while tobacco and cannabis exposure were associated with adolescent and adult tobacco and cannabis use, respectively, prenatal cannabis exposure was also associated with higher rates of tobacco use in a linear dose–response fashion, indicating a non-specific substance effect. In a study examining changes in the association between maternal and child smoking over time, maternal lifetime smoking was associated with adolescent and adult smoking, while prenatal tobacco exposure was not associated with daily smoking in adolescence but was a significant risk factor in young adulthood (Kim et al. 2021), suggesting potential genetic, teratogenic, and environmental effects.

Few studies with a multimethod assessment of smoking and child outcomes use genetically informative designs, although results from behavior genetics suggest these influences may increase with age as individuals evoke responses from others and actively select their environments, explained by active and evocative gene–environment correlation (e.g., McGue & Bouchard 1998). Thus, it is important to consider whether these potentially delayed increasing prenatal exposure effects could be due to genetic associations in addition to teratogenic effects.

### Prenatal Cocaine Exposure

Cocaine-exposed infants exhibit more state lability, orienting, and attention problems in the neonatal period, higher negative affect in response to novel stimulation or stress, and higher behavioral reactivity (shorter latency to anger) in response to frustration with no increases in number of regulatory strategies as stress increased, unlike comparison infants (Eiden et al. 2009). Cocaine exposure is also associated with higher impulsivity at toddler age, lower behavioral regulation during testing, higher frustration and more disruptive behavior during problem-solving tasks at preschool age, poor motor inhibition at school age, higher disruptive behaviors in the school setting (see Eiden et al. 2009), and lower emotion regulation in adulthood (Richardson et al. 2019). There is some evidence of direct, modest associations between amount of cocaine exposure and externalizing problems into adolescence (Min et al. 2014). However, other studies report no direct associations between cocaine exposure and child behavior problems (Accornero et al. 2006) but instead patterns of complex interactions between biological vulnerability and contextual risks from toddler to school age (Eiden et al. 2011a, Molnar et al. 2014), moderation by sex with significantly higher teacher-reported externalizing problems for boys only (Delaney-Black et al. 2011), mediation via higher caregiver harshness to behavior problems (Eiden et al. 2011a), and cascading effects from substance exposure, to lower caregiver sensitivity in early childhood, to lower self-regulation at school age, to higher conduct problems in middle childhood (Ettekal et al. 2020). In well-designed prospective studies controlling for multiple co-occurring risks, cocaine exposure also accounted for unique variance in the early initiation of substance use; higher risk for alcohol, marijuana, and tobacco use; and higher substance-related problems, with cascading (but not direct) effects on substance use disorders in young adulthood (Min et al. 2023). Future studies of substance-exposed samples may examine codevelopment of internalizing and externalizing problems as a function of prenatal substance exposure as well as the role of both dimensions of behavior problems and rearing environment for adolescent risk behaviors (e.g., Marceau et al. 2021).

### Prenatal Exposure to Other Substances and Polysubstance Exposure

Both cocaine and opioids are markers for polysubstance exposure. There is a growing literature on associations between opioids and child behavioral outcomes (for a review, see Schuetze et al. 2021) as well as models examining prenatal exposure as a cumulative proportion risk score, which maintains information about dosage or rank ordering by severity of exposure (e.g., Schuetze et al. 2020b). In their review, Schuetze and colleagues (2021) concluded that despite a number of studies examining child dysregulation and externalizing problems among children with opioid exposure, definitive conclusions are difficult given variations in polysubstance use, methods, and sample sizes. However, there is increasing attention to the issues of polysubstance use, including high prevalence (Tran et al. 2023), and results from multisite cohort studies, such as the Healthy Brain and Child Development (Jordan et al. 2020) study, will likely provide invaluable data regarding brain–behavior outcomes for these children.

## CONTEXTUAL FACTORS

With a few exceptions, the majority of well-designed prospective cohort studies examining the role of prenatal substance exposure for child outcomes do not consider the multitude of co-occurring risks as mediators or moderators using a developmental framework but rather as confounding variables that are statistically controlled in analytic models (Schuetze et al. 2021). While this is important for examining potential teratogenic or substance-specific effects and may have implications for policy and treatment (e.g., specific OAT for opioid use disorder), it does not reflect the complexities of developmental processes that may impact children exposed to substances prenatally. These include higher risk for exposure to family and community violence; caregiver psychological distress and trauma experiences; changes in custody, placement, and caregiving situations; low partner and family support; higher disparities related to income, race, and geographical location; and continued postnatal substance exposure (Schuetze et al. 2021). These risk factors along with substance use itself may interfere with optimal parenting (Schuetze et al. 2021). Below, we briefly review the literature on associations between prenatal substance exposure and parenting, as some of the most proximal mediators or moderators of exposure to child outcome association.

### Parenting

Substances such as cocaine and opioids may interfere with the onset of parenting behaviors and increase risk for low sensitivity during parent–child interactions via effects on hormones such as oxytocin (e.g., Daigle et al. 2020). In addition, the reward–stress dysregulation model of parenting (Rutherford et al. 2013) suggests that substance use reduces the salience and rewarding effects of parent–child interactions via reward-related neural processes, and there is growing evidence of prenatal substance exposure effects on alterations in maternal brain reward processes (Schuetze et al. 2021). Regarding specific substances, the literature on prenatal alcohol exposure and parenting is sparse, although there is a larger literature linking paternal (with some maternal) postnatal alcohol problems and parent–child interaction quality including via comorbid risk factors such as parent antisocial behaviors, depressive symptoms, and couple relationship quality (Eiden et al. 2016, Eiden 2018). The same is true for prenatal tobacco and cannabis exposure, with a few exceptions. Results from the GUH study indicated associations between tobacco exposure and higher maternal anger and depressive symptoms in the prenatal (Eiden et al. 2011b) and postnatal (Ostlund et al. 2021, Schuetze et al. 2019) periods, with significant cascading effects to lower maternal sensitivity in early childhood (Godleski et al. 2016). Little is known about cannabis effects on parent–child interactions, although a small literature indicates more robust associations between tobacco and cannabis co-use and maternal affective dysregulation and/or psychological distress in the prenatal-to-postpartum period (Godleski et al. 2018), with prospective linkages with lower maternal sensitivity during mother–child interactions across the infant–toddler period compared with tobacco use alone (Eiden et al. 2018a). There is a larger literature linking prenatal cocaine exposure to parenting difficulties (Eiden et al. 2023), with human (Light et al. 2004) and animal studies indicating cocaine effects on lower maternal postpartum oxytocin as a putative biological mechanism for this association, as noted above (McMurray et al. 2008). Larger amounts of cocaine may also have an acute effect on increasing aggression, which may have cascading effects on parenting (Eiden et al. 2011d). There is some indication of child evocative effects with prospective indirect associations between prenatal cocaine exposure and less optimal parenting behavior through cocaine’s effects on child behavioral and autonomic regulation (Eiden et al. 2011d,e) as well as moderation of cocaine to parenting associations by infant behavioral reactivity (Eiden et al. 2011e). Results from the MCHS highlight the importance of caregiver negative affect or harshness and low sensitivity as significant mediators of child self-regulatory and externalizing outcomes from preschool to middle childhood in cocaine-exposed samples (Eiden et al. 2015, 2020) and bullying or victimization into late adolescence (Ostrov et al. 2022). Parenting also had additive effects with cocaine exposure on child internalizing problems (Eiden et al. 2014). It is important to note that the literature is by no means unequivocal. Several studies have noted no associations between maternal cocaine use and the quality of mother–child interactions (e.g., Black et al. 1993). These mixed results may be a function of differences in the timing of measurement, varying sample sizes, large within-group heterogeneity on variables such as other substance exposures, placement in foster care, and additional caregiver–child risks or protective factors. Finally, there are few prospective, well-designed, adequately powered studies with prenatal opioid and other substance exposure (for a review, see Schuetze et al. 2021), although there is a growing literature highlighting the important protective effect of the mother–child relationship for opioid-exposed neonates. For instance, studies suggest that rooming-in programs (fostering closeness between mother–infant dyads), compared with separation, decrease the need for pharmacotherapy for opioid-exposed infants and reduce hospital stay length (Newman et al. 2022). In addition, preliminary evidence from ongoing randomized clinical trials (e.g., https://clinicaltrials.gov identifier NCT03891628) (Labella et al. 2021) indicate promising effects of an early childhood parenting program on promoting healthier infant autonomic regulation for opioid-exposed neonates (Tabachnick et al. 2022). These results highlight the role of positive parent–child relationships as potential protective influences, even in the context of parental substance use disorders.

### Other Risk and Protective Factors

A growing body of literature also highlights the importance of continued postnatal exposure or chronicity of exposure across development. Maternal substance use generally decreases after pregnancy recognition, with increases after birth (Eiden et al. 2013, Shisler et al. 2015). Changes in caregiver use across development may be informative beyond the examination of potential teratogenic effects from prenatal exposure. For instance, maternal tobacco and cannabis co-use in the prenatal-to-preschool period, but reduced use after this time point, was associated with offspring co-use patterns in young adulthood (De Genna et al. 2018), highlighting the importance of prenatal-to-early-childhood exposure as a particularly sensitive period. Similarly, chronicity of maternal cannabis use across the pre- to postnatal period was associated with risky sexual behaviors in adolescence (De Genna et al. 2015).

In addition to continued substance exposure, other risk factors noted above often co-occur, and one approach for examining co-occurring risks may be to use a cumulative risk approach (Evans et al. 2013). For instance, results from the MCHS indicate additive effects of prenatal cocaine exposure and cumulative early life adversity (composite of caregiver psychological distress, violence exposure, separations from caregivers, and nonoptimal caregiving routines) on externalizing problem trajectories from toddler to early school age (Molnar et al. 2014); an interaction of adversity and caregiver sensitivity on blunted cortisol responses to social stress in adolescence such that in the context of adversity, sensitive caregiving was protective (Eiden et al. 2023); and four profiles of varying levels of exposure to caregiver substance use, co-occurring family risks, and parenting from infancy to early school age, the majority of children experiencing substantial stability in these profile memberships, with consequences for adolescent well-being (Seay et al. 2023). These examples highlight the importance of adversities and protective factors embedded in the postnatal environment that may have additive, protective, or mediating associations with outcomes across the life span.

## FUTURE DIRECTIONS

### Developmental Frameworks

**Figure 1:**
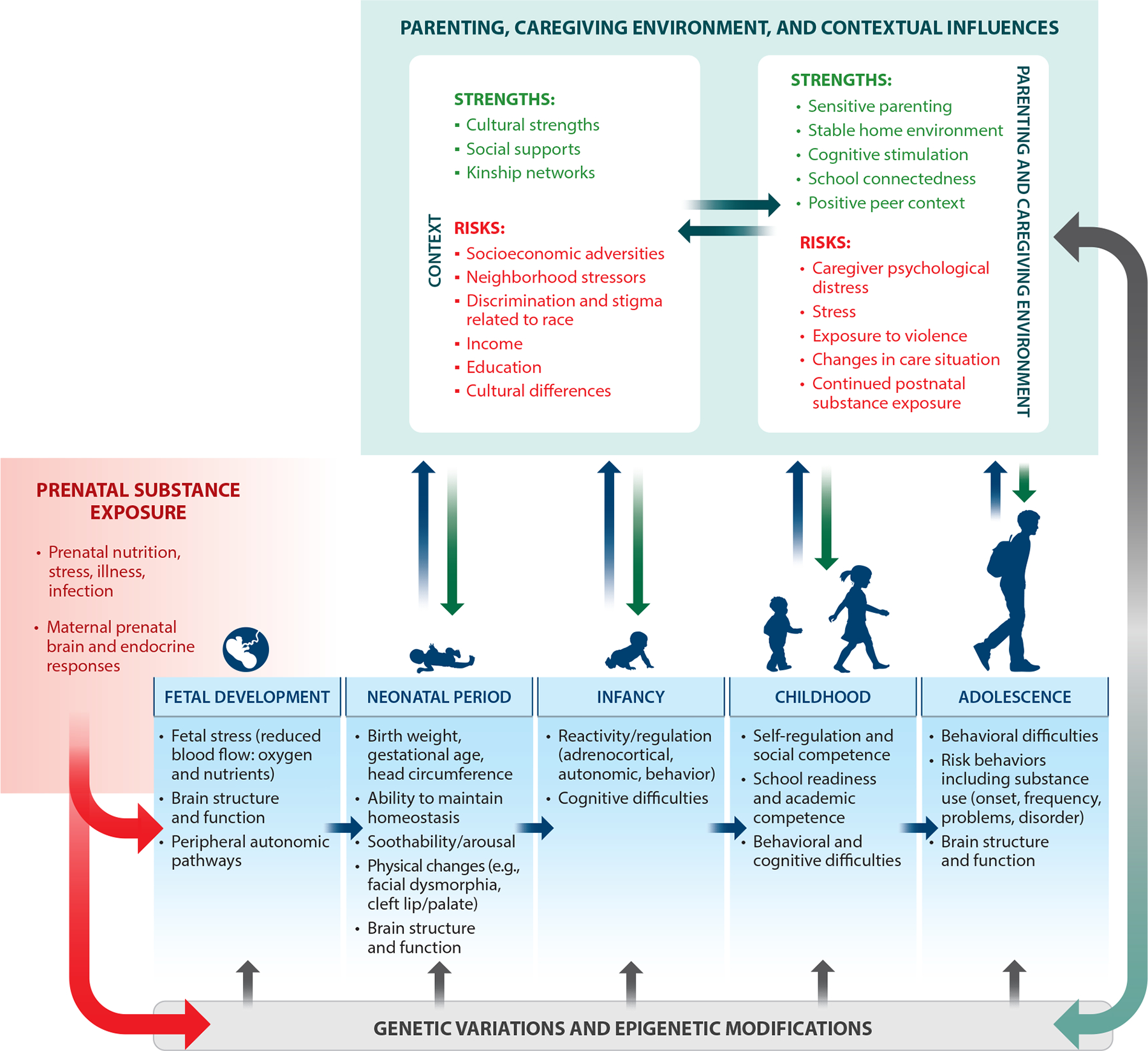
Developmental–transactional model for prenatal substance exposure, co-occurring risks, and postnatal context for key developmental outcomes from fetal period to adolescence.

With increasing acknowledgment and widespread acceptance of the developmental origins of health and disease framework, a greater understanding of the role of epigenetics for lifelong health, and the development and embedding of prenatal substance exposure within a prenatal stress framework, there is greater interest in and acknowledgment of prenatal substance exposure effects by developmental scientists (Horn et al. 2018, Huizink & de Rooij 2018). At the same time, there have been recent calls for the investigation of prenatal stressors to be broader and include co-occurring risks and family processes and to address heterogeneity in outcomes (Huizink & de Rooij 2018). Alternate frameworks that consider genetic (e.g., Estabrook et al. 2016) and environmental (e.g., Eiden et al. 2023) influences, bidirectional associations between parent and child across time, and evocative child effects may be particularly helpful (e.g., Eiden et al. 2018b). Careful consideration of the rearing environment including continued postnatal substance exposure, the role of co-occurring risk factors, and potential protective processes as significant predictors (as opposed to control variables) is critical to move the field forward. To the extent that longitudinal cohorts of substance-exposed families include families facing additional disparities related to race and color, theories that integrate race, ethnicity, and cultural processes in developmental frameworks (Witherspoon & Stein 2023) are important and seldom utilized in this field. Understanding prenatal substance exposure effects on the life span necessitates that our frameworks incorporate lived experiences of families beyond the prenatal period while maintaining high methodological rigor in the measurement of exposure itself and understanding the biology of prenatal exposure on the developing fetus (see Figure 1).

### Multimethod Assessment

The majority of studies on prenatal substance exposure utilize retrospective self-report measures based on one or a few items. Multimethod assessments of exposure that measure changes in use during pregnancy and consider timing, dose, and duration of exposure are needed. For instance, the majority of studies on prenatal tobacco exposure have used retrospective and/or one- or two-item self-report exposure measures. On the basis of such measures, not only were 15–25% of women misclassified as nonsmokers, but also these measures were not predictive of fetal growth outcomes, unlike more intensive, calendar-based self-report measures, biomarkers collected in each trimester of pregnancy, or fetal biomarkers at delivery (Shisler et al. 2017). Indeed, results from this study indicated that the strongest associations between prenatal tobacco exposure and fetal growth were obtained when prenatal exposure was assessed using nicotine and its metabolites in infant meconium. However, measurement based on biomarkers alone may be insufficient to capture first-trimester exposure when use is often at the highest levels, since this includes the time before pregnancy recognition—a marker for significant decreases in use (Eiden et al. 2013).

### Research Designs

Cohort studies beginning in pregnancy that have appropriate comparison groups of demographically similar families or studies using propensity score matching for group comparisons remain limited. Most studies of prenatal substance exposure are cross-sectional, precluding understanding of developmental processes, change over time, sleeper effects, and sensitive periods. For instance, most research on prenatal substance exposure effects on neural development has been conducted in adolescence (Morie et al. 2019), and it is not clear how altered connectivity in infancy might relate to fMRI findings later in development, particularly given methodological limitations of retrospective measures and inadequate comparison groups (Lowell et al. 2022). Indeed, the majority of studies in this area do not have acceptable comparison groups that consider the role of systemic co-occurring risks or cultural and familial strengths on developmental outcomes (Schuetze et al. 2021).

### Sex Differences

We need large cohort studies that are adequately powered to examine moderation by child sex at birth and the role of gender. Moderation of exposure effects by child sex at birth has been reported in several studies, but only a limited number of exposure studies have included such analyses, and fewer are adequately powered to examine moderation (Coles et al. 2012). Thus, it is not surprising that results are mixed, with some studies reporting significant moderation effects while others do not. Variations among studies may be due to all of the methodological issues discussed above but may also be due to the lack of statistical power, developmental timing, and measurement of the outcome.

## CONCLUSION

In conclusion, the role of prenatal substance exposure for child outcomes is complex and the literature is fraught with conceptual and methodological limitations. However, despite these limitations, it is clear that prenatal substance exposure is a marker for increased risk for developmental outcomes but can be understood only in the context of multiple risk and protective factors transacting in the environment. This field is also marked by legal and ethical considerations that vary across regions. How these variations impact development among families with substance use disorders is not well understood and seldom incorporated into exposure studies. Future studies with large sample sizes reflecting the diversity of experiences of children and families experiencing substance exposure, prospective designs, adequate measurement of exposure, appropriate comparison groups, and conceptual frameworks that address the complexities of development are needed to move this field forward.

## Figures and Tables

**Table 1:** Terms and definitions

Term	Definition
Genetically informed designs	Quasi-experimental designs such as sibling comparison and children-of-twin designs. Sibling comparison design is a type of quasi-experimental design where siblings have different levels of prenatal substance exposure (e.g., one sibling has more and one sibling has less substance exposure). This design may control for maternal characteristics and genetic risks when examining exposure effects. A children-of-twin design allows for the examination of bidirectional parent–child influences while accounting for the genetics of both parents and children (passive gene–environment correlation) and shared family environments. These designs allow for the consideration of both genetic and environmental confounds when examining prenatal exposure effects.
Individual participant meta-analysis	Combining original data collected across multiple cohorts or study samples into a single data set for reanalysis and meta-analysis, thereby achieving greater power, the use of the same analytic method for all data, and the ability to conduct more complex, nuanced analyses that may not have been possible due to power considerations within the individual cohorts. This technique also controls for the hierarchical nature of participants nested within a cohort to characterize the impact of prenatal substance exposure across a wider array of participants.
Prospective cohort design	A study design where participants are assessed over time. The sample often includes demographically similar participants who may vary with respect to a key construct. In prenatal substance exposure research, participants may be matched on important demographic characteristics such as age and socioeconomic status but differ on whether they use substances during pregnancy and/or on the type of substance used.
Cross-sectional design	A study design where all data are collected from participants at one time point. These studies often include retrospective reporting of prenatal substance exposure.
Teratogen	An agent that causes abnormalities after fetal exposure.
Quasi-experimental design	A study design focused on identifying cause and effect relationships, but with experimental groups not randomly assigned. This design mimics an experimental design without randomization, usually due to the ethical implications of randomizing the independent variable.
Dose–response	The association between amount of substance exposure (e.g., number of cigarettes per day, standard drinks per day) during pregnancy and an outcome.
Case–control	Designs where participants are recruited into two or more groups on the basis of substance exposure status with cases including those with substance exposure (e.g., cocaine) and controls including participants without exposure to that specific substance (non-cocaine). Control groups may include use of other substances (e.g., alcohol, cigarettes, cannabis) to examine if cocaine exposure (which mostly occurs in the context of polysubstance use) accounts for unique variance in outcomes and/or may include participants with no substance exposure (to examine effects of polysubstance exposure including cocaine). The nature of the control group is an important consideration based on the research question of interest. Control groups also allow for matching of participants on key demographic variables (e.g., socioeconomic status).
